# Caesarean section defects may affect pregnancy outcomes after in vitro fertilization-embryo transfer: a retrospective study

**DOI:** 10.1186/s12884-021-03955-7

**Published:** 2021-07-06

**Authors:** Junrong Diao, Ge Gao, Yunshan Zhang, Xinyan Wang, Yinfeng Zhang, Ying Han, Aijun Du, Haining Luo

**Affiliations:** Center for Reproductive Medicine, Tianjin Central Hospital of Obstetrics and Gynecology, No. 156 Nankai San Ma Road, Tianjin, 300000 China

**Keywords:** Caesarean section defect, In vitro fertilization-embryo transfer, Pregnancy

## Abstract

**Background:**

Caesarean section rates are rising worldwide. One adverse effect of caesarean section reported in some studies is an increased risk of subfertility. Only a few studies have assessed the relationship between the previous mode of delivery and in vitro fertilization/intracytoplasmic sperm injection-embryo transfer (IVF/ICSI-ET) reproductive outcomes. In this study, we primarily investigated the impact of a history of caesarean section with or without defects on IVF/ICSI-ET outcomes compared to a vaginal delivery history.

**Methods:**

This retrospective study included 834 women who had a IVF or ICSI treatment at our centre between 2015 and 2019 with a delivery history. In total, 401 women with a previous vaginal delivery (VD) were assigned to the VD group, and 433 women with a history of delivery by caesarean section were included, among whom 359 had a caesarean scar (CS) without a defect and were assigned to the CS group and 74 had a caesarean section defect (CSD) and were assigned to the CSD group. Baseline characteristics of the three groups were compared and analysed. Binary logistic regression analyses were performed to explore the association between clinical outcomes and different delivery modes.

**Results:**

There were no significant differences in the live birth rate, biochemical pregnancy rate, clinical pregnancy rate, mean implantation rate or abnormal pregnancy rate between the CS and VD groups However, the live birth rate and mean implantation rate in the CSD group were significantly lower than those in the VD group (21.6 *vs* 36.4%, adjusted OR 0.50 [0.27–0.9]; 0.25 ± 0.39 *vs* 0.35 ± 0.41, adjusted OR 0.90 [0.81–0.99]). Among women aged ≤ 35 years, the subgroup analyses showed that the live birth rate, biochemical pregnancy rate, clinical pregnancy rate, and mean implantation rate in the CSD group were all significantly lower than those in the VD group (21.4 *vs* 45.8%, adjusted OR 0.35[0.15 ~ 0.85]; 38.1 *vs* 59.8%, adjusted OR 0.52[0.24–0.82]; 31.0 *vs* 55.6%, adjusted OR 0.43[0.19–0.92]; 0.27 ± 0.43 *vs* 0.43 ± 0.43, adjusted OR 0.85[0.43 ± 0.43]). For women older than 35 years, there was no statistically significant difference in any pregnancy outcome among the three groups.

**Conclusions:**

This study suggested that the existence of a CS without a defect does not decrease the live birth rate after IVF or ICSI compared with a previous VD. However, the presence of a CSD in women, especially young women (age ≤ 35 years), significantly impaired the chances of subsequent pregnancy.

## Introduction

Caesarean section rates have continued to rise worldwide. Recently, the Lancet conducted a global statistical analysis on caesarean sections and reported that from 2000 to 2015, the global caesarean section rate almost doubled, from 12 to 21%, with rates of 32.8% in the United States, 32.4% in Australia and 36.2% in China [1]. This rise was due mainly to the expansion of caesarean section indications, a dramatic decline in vaginal delivery rates after a previous caesarean section and an increased maternal demand for the procedure [2–4].

This continuously growing trend has led to an interest in potential long-term obstetric sequelae after caesarean section. The well-known obstetric complications in subsequent pregnancies include caesarean scar pregnancy, placental abnormalities, uterine rupture and preterm birth [5–8]. In addition, adverse effects of caesarean section, reported in some retrospective and observational studies, could increase the risk of subfertility [9, 10]. A large meta-analysis, including 16 studies, reported that a history of caesarean section reduced the chances of a subsequent pregnancy by 9% compared with a vaginal delivery [11]. A retrospective case–control study including 310 IVF patients showed that a caesarean section scar can could decrease the chances of embryo implantation (24.01 *vs*. 34.67%) and reduce the pregnancy rate (40.28 *vs*. 54.22%) [12]. However, the studies by Zhang N et al. (a retrospective study of 231 IVF patients) and Patounakis G et al. (a single-site prospective cohort study of 194 IVF patients) reported that women with a previous caesarean section had embryo implantation and pregnancy rates similar to those of women with prior vaginal deliveries [13, 14]. Therefore, the detrimental effect of caesarean section on IVF outcomes is uncertain.

A caesarean section defect (CSD, also called a niche) is defined as a disruption of the integrity of the myometrium at the site of the uterine scars.. Niches are observed in 24–70% of women after a caesarean section when assessed by transvaginal sonography (TVS) [15]. It is reported to be associated with detrimental gynaecological symptoms, such as abnormal uterine bleeding, pelvic pain, and infertility [16, 17]. Naji et al. suggested that embryo implantation near or in the caesarean scar defect may result in a higher miscarriage rate [18]. In the study by Wang et al., it was reported that the pregnancy rate sharply declines if a CSD is present [12]. The existence of the CSD may be one of the main reasons for subfertility among women with a previous caesarean section. To date, no study has previously evaluated the relationship between the presence of a defect and the reproductive outcomes of women undergoing IVF or ICSI treatment.

The abolition of the single-child policy in China has contributed to a marked increase in the number of infertility patients with a history of caesarean section who had to choose IVF/ICSI-ET for conceiving a second child. It is of major importance to investigate the effects of a history of caesarean section on women receiving IVF treatment. The aim of this retrospective study was to evaluate the relationship between caesarean section scars with or without defects and reproductive outcomes among women after IVF/ICSI-ET.

## Methods

### Patients

In this retrospective study, all women with secondary infertility and a history of delivery who underwent IVF/ICSI-ET treatment at our IVF centre between January 2015 and December 2019 were included. The inclusion criteria were as follows: (1) patients had a history of a previous delivery beyond 5 months gestation; (2) treatment with either a long mid-luteal GnRH antagonist or GnRH antagonist ovarian stimulation protocol; (3) undergoing a first fresh embryo transfer; and (4) complete clinical data and follow-up records. The exclusion criteria were as follows: (1) a history of myomectomy or resection of an endometrioma; (2) congenital or acquired uterine malformations, uterine fibroids, adenomyosis, or intrauterine adhesion; (3) abnormal results on parental karyotyping; (4) recurrent spontaneous abortion history (defined as two or more previous spontaneous pregnancy losses); or (5) chromosomal abnormalities.

According to the above inclusion and exclusion criteria, a total of 834 women were enrolled in this study. Depending on the mode of delivery and the presence of a CSD, all women were divided into three groups: the vaginal delivery (VD) group (*n* = 401), caesarean scar without a defect (CS) group (*n* = 359) and CSD group (*n* = 74). Women were further divided into subgroups based on age (≤ 35 and > 35 years). According to the presence of fluid in the niche or endometrial cavity, patients in the CSD group were divided into two small subgroups: the non-cavity fluid group (*n* = 49) and the cavity fluid group (*n* = 25). The same trained sonographer used two-dimensional transvaginal ultrasound (America,GE-Voluson E8) to determine whether a patient had a previous CSD. The diagnostic criterion was a visible defect that demonstrated a triangular anechoic area or a fluid-filled defect in the uterine scar through TVS.. Transvaginal sonography (TVS) is a non-invasive and effective tool for evaluating the healing of the uterine segment, measuring the size, shape and position of the CSD, and detecting endometrial fluid.

### IVF protocol

Patients underwent controlled ovarian hyperstimulation (COH) with either a long mid-luteal GnRH agonist or GnRH antagonist protocol. Each patient was monitored by transvaginal ultrasound and blood ovarian steroid hormone levels. The endometrial thickness, size of each follicle, number of follicles, and necessary hormone levels were recorded during the process of ovarian stimulation. According to the above parameters, the dosage and time of gonadotropin (Gn) were adjusted. When at least two dominant follicles were 18 mm or larger, human chorionic gonadotropin (hCG) or leuprolide acetate was administered to trigger final oocyte maturation. Patients underwent transvaginal oocyte retrieval (TVOR) 36 h after the trigger. Conventional IVF or ICSI was performed following oocyte retrieval according to the kinetic analysis of the washed sperm suspension. Embryos were cultured in vitro for 2 or 3 days prior to transfer. Normally, no more than two embryos are selected for transfer. In a few exceptional cases, such as when the patient was older or when the embryo quality was poor, three embryos were transferred. Supernumerary embryos were cryopreserved. Patients received luteal support on day 2 or 3 after TVOR in the form of intramuscular progesterone 80 mg/day or intravaginal progesterone gel 90 mg/day. Patients receiving intramuscular progesterone should be instructed to continue 80 mg/day until the pregnancy test is positive and then gradually reduce the dose according to the pregnancy status.

High-quality embryos were defined as normal fertilized embryos with 6–10 regular blastomeres and < 10% fragmentation on day 3 [19].

### Pregnancy follow-up

A biochemical pregnancy test (positive hCG test) was initially conducted 14 days after ET. Transvaginal ultrasound was performed between 6–7 weeks of gestation to confirm clinical pregnancy by observing the presence of a gestational sac with or without foetal heartbeat. Information on subsequent pregnancy outcomes was collected through telephone-interview by clinicians and nurses.

### Observation indicators

Basic information about the patients, including age, infertility duration, infertility cause, infertility category (primary/secondary), body mass index (BMI), baseline follicle-stimulating hormone (FSH), Gn dose, duration of stimulation, oestradiol levels on the trigger day, endometrial thickness on the trigger day, and number of follicles, was recorded. The primary outcome measure was the live birth rate. Prespecified secondary outcomes included the positive hCG test rate, clinical pregnancy rate, mean implantation rate, abnormal pregnancy rate, mode of delivery, and premature birth rate.

A positive hCG was defined as a value greater than 5 IU/L. Clinical pregnancy was defined as the presence of a gestational sac seen on transvaginal ultrasound. The mean implantation rate was defined as the ratio of the number of amniotic sacs per patient to the number of embryos transferred per patient. Ectopic pregnancy was defined as a clinical pregnancy outside the uterus. Early abortion referred to spontaneously aborted pregnancy before the gestational age of 12 weeks. Late abortion referred to spontaneously eventuated pregnancy between 12 and 28 weeks of gestation. Mid-term induction refers to the artificial termination of pregnancy between 12 and 28 weeks of gestation due to maternal and foetal reasons. Live birth refers to all live births.

### Statistical analysis

Continuous variables are presented as the mean ± standard deviation or median (lower quartile, upper quartile) according to data distribution. Categorical variables are presented as numbers with percentages. The SNK-q test, Kruskal–Wallis H test and Pearson’s χ2 test were used to compare baseline characteristics among the three groups as appropriate. As outcome indicators included dichotomous or continuous variables, binary logistic regression analysis and generalized linear models were used as appropriate to test the relationship between the mode of previous delivery and reproductive outcomes. Crude and adjusted odds ratios (ORs) with 95% confidence intervals (CIs) were calculated before and after adjusting for confounding variables, including age, infertility cause, infertility category, BMI, endometrial thickness on the trigger day, fertilization methods, number of embryos transferred, number of high-quality embryos transferred and day of transfer. Statistical analysis was performed with SPSS (Version 20.0. SPSS, Inc., Chicago, IL, USA). All tests were two-sided, and P < 0.05 was considered statistically significant. The statistical power was calculated using PASS 11 (Hintze, J. (2011). PASS 11. NCSS, LLC. Kaysville, UT, USA; www.ncss.com).

## Results

### General patient information

A total of 843 patients were included in this study. The baseline characteristics of the women in the three groups are shown in Table 1. General characteristics such as age, infertility cause, infertility category, BMI, infertility duration, and type of stimulation protocol were not significantly different among the three groups. There were no statistically significant differences in ovarian response-related factors, such as baseline FSH, mean E_2_ level on the trigger day, dosage of Gn, duration of Gn stimulation, and number of follicles with a diameter ≥ 14 mm on the trigger day, indicating that the presence of a previous caesarean section scar with or without a defect did not impact ovarian reserve function or the ovarian response. Statistically significant differences were not observed for embryo development-related factors, such as the number of retrieved oocytes, normal fertilization rate, cleavage rate, number of available embryos, and number of high-quality embryos, among the three groups. These results showed that a previous caesarean section did not affect fertilization or early embryo development. Furthermore, there were no differences in fertilization methods or the number of embryos transferred among the three groups. However, endometrial thickness on the trigger day, in the CSD group was significantly lower than that in the other two groups (9.87 ± 1.74 *vs* 9.72 ± 1.82 *vs* 9.28 ± 1.50, *P* < 0.05), and there were statistically significant differences in the number of high-quality embryos transferred among the three groups [1 (1,2) *vs* 1 (1,1) *vs* 1 (1,1), *P* < 0.05]. The proportion of D2 embryos transferred in the CSD group was significantly higher than that in the other two groups (16.2 *vs* 8.2 *vs* 5.3%, *P* < 0.05).

**Table 1:** Baseline characteristics of of study participants according to by previous delivery modes

**Parameter**	**VD group (*n* = 401)**	**CS group (*n* = 359)**	**CSD group (*n* = 74)**	***P***
Age (years)	35.05 ± 4.51	35.11 ± 3.97	34.53 ± 4.04	0.552
Infertility duration (years)	4.71 ± 3.60	4.14 ± 3.13	4.14 ± 2.93	0.051
Infertility cause, n(%)				
Tubal factor	199(49.6)	164(45.7)	34(45.9)	0.278
Ovary factor	46(11.5)	44(12.3)	12(16.2)	
Male factor	60(15.0)	38(10.6)	7(9.5)	
Unexplained	24(6.0)	24(6.7)	4(5.4)	
Combined	72(18.0)	89(24.8)	17(23.0)	
Infertility category, n(%)				
Primary	10(2.5%)	11(3.1%)	3(4.1%)	0.744
Secondary	391(97.5%)	348(96.9%)	71(95.9%)	
BMI (kg/m^2^)	23.21 ± 2.87	23.50 ± 3.11	23.14 ± 2.83	0.347
Basal FSH (IU/L)	7.00 ± 2.45	6.81 ± 2.39	7.39 ± 2.99	0.158
Stimulation protocol, n(%)				
Md-luteal Lupron	252(62.8)	227(63.2)	45(60.8)	0.926
Antagonist	149(37.2)	132(36.8)	29(39.2)	
Total gonadotropins (IU)	2951.35 ± 774.28	2914.25 ± 700.88	2960.51 ± 697.32	0.753
Duration of stimulation (days)	9.23 ± 1.60	9.25 ± 1.60	9.23 ± 1.40	0.990
E_2_ level on the trigger day (pg/mL)	3159.21 ± 1928.37	3104.15 ± 1785.13	3127.23 ± 1693.28	0.919
Endometrial thickness on the trigger day (mm)	9.87 ± 1.74	9.72 ± 1.82	9.28 ± 1.50	0.026
Numbers of follicles with a diameter ≥ 14 mm on the day of trigger	10.80 ± 5.27	11.34 ± 5.38	10.76 ± 4.48	0.328
Retrieved oocytes (n)	10.10 ± 5.60	10.72 ± 5.56	10.15 ± 5.01	0.281
Fertilization methods, n(%)				
IVF	266(66.3)	244(68.0)	53(71.6)	0.651
ICSI	135(33.7)	115(32.0)	21(28.4)	
Fertilization rate (%)	76.9(2683/3491)	76.6(2570/787)	77.6(502/647)	0.843
Cleavage rate (%)	98.4(2640/2683)	98.2(2524/2570)	98.6(495/502)	0.767
Available embryos(n)	4(2,6)	4(2,6)	3(2,6)	0.584
High-quality embryos(n)	2(1,4)	2(1,4)	2(1,4)	0.448
Number of embryos transferred (n)	2(1,2)	1(1,2)	1(1,2)	0.087
Day of Transfer, n(%)				
D2	33(8.2%)	19(5.3%)	12(16.2%)	0.005
D3	368 (91.8%)	340(94.7%)	62(83.8%)	
Number of high-quality embryo transferred(n)	1(1,2)	1(1,1)^a^	1(1,1)	0.000

### Pregnancy outcomes after IVF/ICSI-ET

**Fig. 1:**
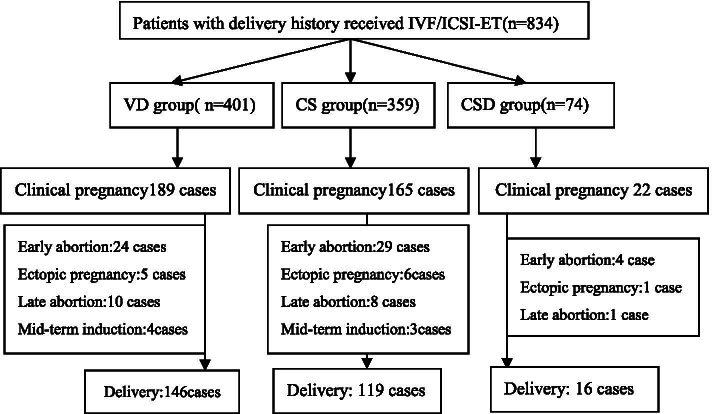
Flowchart of reproductive outcomes after IVF/ICSI-ET

The post-IVF/ICSI-ET pregnancy outcomes of the three groups are shown in Fig. 1.

Binary logistic regression analysis and generalized linear models were performed to determine the effect of a history of caesarean section on pregnancy outcomes. Table 2, presents the results of both the crude analyses and the analyses adjusted for age, infertility cause, infertility category, BMI, endometrial thickness on the trigger day, fertilization methods, number of embryos transferred, number of high-quality embryos transferred and day of transfer. There were no significant differences in the live birth rate, biochemical pregnancy rate, clinical pregnancy rate, mean implantation rate or abnormal pregnancy rate between the CS and VD groups (*P* > 0.05). However, the live birth rate in the CSD group was significantly lower than that in the VD group (21.6 *vs* 36.4%, crude OR 0.48 [0.26–0.86]; adjusted OR 0.50 [0.27–0.95]). The mean implantation rate was also significantly lower in the CSD group than in the VD group after adjusting for confounding factors (0.35 ± 0.41 *vs* 0.25 ± 0.39, adjusted OR, 95% CI 0.90 [0.81 ~ 0.99]). Positive hCG test and clinical pregnancy rates were also lower in the CSD group before correction for confounding factors (35.1 *vs* 51.4%, OR 0.51[0.30–0.85]; 29.7 *vs* 47.1%, OR 0.47[0.27–0.8]). When the confounding variables were added to the logistic regression models, there was no longer an association between prior CSD and pregnancy outcomes, with adjusted ORs of 0.67 (0.39–1.18) and 0.59 (0.33–1.06) for the positive hCG test and clinical pregnancy rates, respectively. Compared with those in the previous vaginal delivery group, the early abortion and ectopic pregnancy rates were not significantly increased in the CSD group.

**Table 2:** Binary logistic regression analysis

**Parameter**	**%(n/N)**	**Crude OR (95%CI)**	**Adjusted OR (95%CI)**
Live birth			
VD (*n* = 401)	36.4%(146/401)	1.00	1.00
CS (*n* = 359)	33.1%(119/359)	0.86(0.64 ~ 1.16)	0.93(0.67 ~ 1.29)
CSD (*n* = 74)	21.6%(16/74)	0.48(0.26 ~ 0.86)*	0.50(0.27 ~ 0.95)*
Positive hCG test			
VD (*n* = 401)	51.4%(206/401)	1.00	1.00
CS (*n* = 359)	50.1%(180/359)	0.95(0.71 ~ 1.26)	1.16(0.84 ~ 1.59)
CSD (*n* = 74)	35.1%(26/74)	0.51(0.30 ~ 0.85)*	0.67(0.39 ~ 1.18)
Clinical pregnancy			
VD (*n* = 401)	47.1%(189/401)	1.00	1.00
CS (*n* = 359)	46.0%(165/359)	0.95(0.71 ~ 1.26)	1.13(0.82 ~ 1.56)
CSD (*n* = 74)	29.7%(22/74)	0.47(0.27 ~ 0.81)*	0.59(0.33 ~ 1.06)
Mean implantation rate (mean ± SD)			
VD (*n* = 401)	0.35 ± 0.41	1.00	1.00
CS (*n* = 359)	0.37 ± 0.44	1.02(0.96 ~ 1.09)	1.01(0.95 ~ 1.08)
CSD (*n* = 74)	0.25 ± 0.39	0.90(0.81 ~ 1.004)	0.90(0.81 ~ 0.99)*
Early abortion			
VD (*n* = 401)	12.7%(24/189)	1.00	1.00
CS (*n* = 359)	17.6%(29/165)	1.38(0.78 ~ 2.41)	1.58(086 ~ 2.88)
CSD (*n* = 74)	18.2%(4/22)	0.89(0.30 ~ 2.66)	1.25(0.40 ~ 3.91)
Ectopic pregnancy			
VD (*n* = 401)	2.6%(5/189)	1.00	1.00
CS (*n* = 359)	3.6%(6/165)	1.34(0.40 ~ 4.44)	1.81(0.49 ~ 6.62)
CSD (*n* = 74)	4.5%(1/22)	1.08(0.12 ~ 9.42)	1.76(0.18 ~ 17.18)

^*^P < 0.05 (adjusted for age, BMI, infertility cause, infertility category, endometrial thickness on the trigger day, fertilization methods, the number of embryos transferred, high-quality embryo transferred and day of transfer)

Table 3 presents the results of the crude and adjusted ORs between groups for women aged ≤ 35 years (VD = 214, CS = 187, CSD = 42). Similarly, there were no significant differences in the live birth rate, biochemical pregnancy rate, clinical pregnancy rate, mean implantation rate or abnormal pregnancy rate between the CS and VD groups (*P* > 0.05).However, the live birth rate, positive hCG test rate, clinical pregnancy rate and mean implantation rate in the CSD group were all significantly lower than those in the VD group (21.4 *vs* 45.8%, adjusted OR 0.35 [0.15–0.85]; 38.1 *vs* 59.8%, adjusted OR 0.52 [0.24–0.82]; 31.0 *vs* 55.6%, adjusted OR 0.43 [0.19–0.92]; 0.27 ± 0.43 *vs* 0.43 ± 0.43, adjusted OR 0.85 [0.43 ± 0.43]).

**Table 3:** Binary logistic regression analysis for women aged ≤ 35 years

**Parameter**	**%(n/)**	**Crude OR (95%CI)**	**Adjusted OR (95%CI)**
Live birth			
VD (*n* = 214)	45.8%(98/214)	1.00	1.00
CS (*n* = 187)	37.4%(70/187)	0.70(0.47 ~ 1.05)	0.81(0.52 ~ 1.33)
CSD (*n* = 42)	21.4%(9/42)	0.32(0.14 ~ 0.70)*	0.35(0.15 ~ 0.85)*
Positive hCG test			
VD (*n* = 214)	59.8%(128/214)	1.00	1.00
CS (*n* = 187)	49.2%(92/187)	0.65(0.43 ~ 0.96)	0.82(0.52 ~ 1.27)
CSD (*n* = 42)	38.1%(16/42)	0.41(0.20 ~ 0.81)*	0.52(0.24 ~ 0.82)*
Clinical pregnancy			
VD (*n* = 214)	55.6%(119/214)	1.00	1.00
CS (*n* = 187)	46.0%(86/187)	0.68(0.45 ~ 1.008)	0.84(0.53 ~ 1.33)
CSD (*n* = 42)	31.0%(13/42)	0.35(0.17 ~ 0.72)*	0.43(0.19 ~ 0.92)*
Mean implantation rate (mean ± SD)			
VD (*n* = 214)	0.43 ± 0.43	1.00	1.00
CS (*n* = 187)	0.41 ± 0.47	0.97(0.89 ~ 1.06)	0.97(0.89 ~ 1.06)
CSD (*n* = 42)	0.27 ± 0.43	0.84(0.73 ~ 0.98)*	0.85(0.73 ~ 0.98)*
Early abortion			
VD (*n* = 214)	9.2%(11/119)	1.00	1.00
CS (*n* = 187)	9.3%(8/86)	0.82(0.32 ~ 2.09)	0.97(0.34 ~ 2.76)
CSD (*n* = 42)	15.4%(2/13)	0.92(0.19 ~ 0.19)	1.03(0.20 ~ 5.27)
Ectopic pregnancy			
VD (*n* = 214)	2.5%(3/119)	1.00	1.00
CS (*n* = 187)	2.3%2/86)	0.76(0.12 ~ 4.60)	1.17(0.15 ~ 8.94)
CSD (*n* = 42)	7.7%(1/13)	1.71(0.17 ~ 16.90)	2.94(0.19 ~ 44.65)

^*^P < 0.05 (adjusted for age, BMI, infertility cause, infertility category, endometrial thickness on the trigger day, fertilization methods, the number of embryos transferred, high-quality embryo transferred and day of transfer)

Table 4 presents the results of the crude and adjusted ORs between groups for women aged > 35 years (VD = 187, CS = 172, CSD = 32). There were no statistically significant differences in any pregnancy outcome among the three groups with or without adjustments for various confounders (*P* > 0.05).

**Table 4:** Binary logistic regression analysis for women aged > 35 years

**Parameter**	**%(n/N)**	**Crude OR (95%CI)**	**Adjusted OR (95%CI)**
Live birth			
VD (*n* = 187)	25.7%(48/187)	1.00	1.00
CS (*n* = 172)	28.5%(49/172)	1.15(0.72 ~ 1.83)	1.15(0.69 ~ 1.90)
CSD (*n* = 32)	21.9%(7/32)	0.81(0.33 ~ 1.99)	0.85(0.33 ~ 2.20)
Positive hCG test			
VD (*n* = 187)	41.7%(78/187)	1.00	1.00
CS (*n* = 172)	51.2%(88/172)	1.46(0.96 ~ 2.22)	1.68(1.06 ~ 2.68)
CSD (*n* = 32)	31.2%(10/32)	0.63(0.28 ~ 1.41)	0.88(0.37 ~ 2.07)
Clinical pregnancy			
VD (*n* = 187)	37.4%(70/187)	1.00	1.00
CS (*n* = 172)	45.9%(79/172)	1.42(0.93 ~ 2.16)	1.55(0.97 ~ 2.47)
CSD (*n* = 32)	28.1%(9/32)	0.65(0.28 ~ 1.49)	0.86(0.35 ~ 2.07)
Early abortion			
VD (*n* = 187)	18.6%(13/70)	1.00	1.00
CS (*n* = 172)	26.6%(21/79)	1.86(0.90 ~ 3.84)	2.07(0.94 ~ 4.55)
CSD (*n* = 32)	22.2%(2/9)	0.89(0.19 ~ 4.15)	1.37(0.27 ~ 6.87)
Ectopic pregnancy			
VD (*n* = 187)	2.9%(2/70)	1.00	1.00
CS (*n* = 172)	5.1%(4/79)	2.20(0.39 ~ 12.17)	2.00(0.30 ~ 13.11)
CSD (*n* = 32)	0	NA	NA

(adjusted for age, BMI, infertility cause, infertility category, endometrial thickness on the trigger day, fertilization methods, the number of embryos transferred, high-quality embryo transferred and day of transfer)

Table 5 presents the results of the subgroup analysis of women who did and did not have fluid in the cavity (non-cavity fluid group = 49, cavity fluid group = 25). The live birth rate, biochemical pregnancy rate, clinical pregnancy rate, and mean implantation rate in the cavity fluid group were all lower than those in the non-cavity fluid group. However, there were no statistically significant differences in any pregnancy outcome between the two subgroups before and after adjustment for confounders (*P* > 0.05).

**Table 5:** Subgroup analysis of women who did and did not have fluid in the cavity

Parameter	Non-cavity fluid group (*n* = 49)	Cavity fluid group (*n* = 25)	Crude OR (95%CI)	Adjusted OR (95%CI)
Live birth	22.4%(11/49)	20.0%(5/25)	0.86(0.26 ~ 2.83)	1.06(0.26 ~ 4.34)
Positive hCG test	38.8%(19/49)	28%(7/25)	0.61(0.21 ~ 1.74)	0.76(0.20 ~ 2.85)
Clinical pregnancy	32.7%(16/49)	24%(6/25)	0.65(0.21 ~ 1.94)	0.96(0.24 ~ 3.77)
Mean implantation rate (mean ± SD)	0.26 ± 0.39	0.22 ± 0.41	0.95(0.79 ~ 1.15)	1.01(0.84 ~ 1.20)
Early abortion	4/16	0/6		
Ectopic pregnancy	0/16	1/6		

(adjusted for age, infertility cause, infertility category, BMI, endometrial thickness at the hCG trigger, fertilization methods, the number of embryos transferred, high-quality embryos transferred, and day of transfer)

### Delivery outcomes after IVF/ICSI-ET

The delivery outcomes and neonatal conditions of the three groups after IVF/ICSI-ET are shown in Table 6. We collected information regarding the delivery outcomes of 281 women. Although the previous delivery of each woman was vaginal, the caesarean section rate after IVF/ICSI-ET was 54.1% in the VD group, while in the CS and CSD groups, the caesarean section rates were 95.8 and 93.8%, respectively. The difference in the caesarean section rate among the three groups was statistically significant (*P* < 0.05). The twin delivery rate was 23.3% in the VD group and 6.7% in the CS group, but there was no twin delivery in the CSD group. Eighteen patients with multiple pregnancies underwent selective embryo reduction (10 cases in the VD group, 6 cases in the CS group, and 2 cases in the CSD group). There were no differences in the full-term birth rate, preterm birth rate, birth weight, or low-birth-weight infant rate among the three groups. However, as shown in Table 6, the preterm birth rate was 31.3% in the CSD group, which was higher than that in the VD and CS groups (15.8 and 10.9%, respectively). Two cases of placenta previa occurred in the CS group. One case of placenta previa occurred in the CS group. None of the three groups included women with postpartum haemorrhage or uterine rupture.

**Table 6:** Delivery outcomes and neonatal conditions after embryo transfer

**Parameter**	**VD group (*n* = 146)**	**CS group (*n* = 119)**	**CSD group (*n* = 16)**	***P***
Caesarean delivery rate (%)	54.1%(79/146)	95.8%(114/119)^a^	93.8%(15/16)^b^	0.000
Twin-births rate (%)	23.3%(34/146)	6.7%(8/119)	0	
Full-term birth rate (%)	84.2%(123/146)	89.1%(106/119)	68.7%(11/16)	0.082
Pre-term birth rate (%)	15.8%(23/146)	10.9%(13/119)	31.3%(5/16)	0.082
Birth weight (g)	3104.7 ± 659.88	3227.0 ± 602.92	3231.8 ± 480.15	0.221
Low birth weight rate (%)	13.9%(25/180)	9.4%(12/127)	12.5%(2/16)	0.500

^a^VD group vs CS group, *P* < 0.05; ^b^VD group vs CSD group, *P* < 0.05

## Discussion

This retrospective observational study based on the IVF/ICSI-ET population revealed no differences in pregnancy outcomes of women with only a prior caesarean section without a defect compared to those with a prior vaginal delivery. However, the presence of a CSD in women, especially young women (age ≤ 35 years), significantly impaired the chances of subsequent pregnancy.

The relationship between a history of caesarean section and subsequent fertility is of concern because caesarean section rates continue to rise. Various studies have reported that a prior caesarean section may reduce subsequent fertility rates and prolong pregnancy intervals [20–23]. To date, only a few studies have assessed the association between previous caesarean section and IVF/ICSI-ET pregnancy outcomes [12–14, 24, 25]. Wang et al. conducted a retrospective cohort study of 310 patients undergoing fresh IVF/ICSI cycles [12]. The authors reported lower implantation rates (24.01 *vs.* 34.67%, *P* < 0.05) and clinical pregnancy rates (40.28 *vs.* 54.22%, *P* < 0.05) after caesarean section than after vaginal delivery. In addition, when a CSD in combination with endometrial fluid was present, clinical pregnancy rates sharply decreased to 12.5%. However, one limitation of the study was that it did not adjust various confounding factors on pregnancy outcomes. Vissers J et al. researched 1317 IVF cycles and found that live birth rates were significantly lower among women with a previous caesarean section than among women with a previous vaginal delivery (15.9 versus 23.3%, respectively [OR 0.63 95% CI 0.45–0.87]) [24]. The ongoing pregnancy, clinical pregnancy and biochemical test rates were also lower. Furthermore, the study noted even lower live birth rates after a CSD (10.7%). Unfortunately, the sample size of this subgroup was too small for effective statistical analysis. A more recent study by Huang et al. investigated the effect of a prior caesarean section on pregnancy outcomes of frozen-thawed embryo transfer (FET) in freeze-all cycles. Pregnancy and higher early miscarriage rates were lower among women with a previous caesarean section than among women with a previous vaginal delivery [25].

However, other studies have reported opposite conclusions. The retrospective study by Zhang et al., which included a total of 231 patients*,* showed that a previous caesarean section scar did not affect embryo implantation or pregnancy outcomes after IVF [13]. However, several limitations of this study should be noted, including imbalances in baseline characteristics (maternal age, infertility duration) and uncorrected confounders that affected outcomes. Patounakis et al. conducted a prospective cohort study of 194 patients undergoing fresh IVF/ICSI cycles [14]. The study demonstrated that despite the apparently more difficult transfers, clinical pregnancy (41 *vs.* 49%) and live birth rates (32 *vs.* 39%) were not different between patients with a history of caesarean section and those with only a prior vaginal delivery. The prospective design of the study reduced the biases that affect retrospective studies and provided a higher level of evidence. However, the study stopped before it could enrol the planned number of patients.

In our study, there was no significant difference in the live birth rate between the CS and VD groups (33.1 *vs* 36.4%, OR 0.96 95% CI 0.69–1.34). Despite adjustments for confounders, such as age, infertility cause, infertility category, BMI, endometrial thickness on the trigger day, fertilization methods, number of embryos transferred, number of high-quality embryos transferred and day of transfer, the results remained the same. This showed that the presence of a caesarean section scar alone did not reduce the rates of pregnancy outcomes among IVF cycles. However, the live birth rate was significantly lower among women with a previous CSD, at 21.6%, than among women with a previous vaginal delivery, at 36.4% (OR 0.50 95% CI 0.27–0.95). The mean implantation rate was also significantly lower in the CSD group than in the VD group after adjusting for confounding factors (0.35 ± 0.41 *vs* 0.25 ± 0.39, respectively, OR 0.50, 95% CI 0.27–0.95). Furthermore, among females aged ≤ 35 years, the live birth rate decreased sharply with the existence of a niche, compared with women with a previous vaginal delivery (21.4 versus 45.8%, OR 0.35 95% CI 0.15–0.85). Additionally, the positive hCG test rate, clinical pregnancy rate and mean implantation rate were also significantly reduced among women with a niche. These results indicate that a uterine defect may have a detrimental effect on IVF/ICSI-ET outcomes. In young females, in particular, the negative effects were more prominent. However, for females over 35 years old, the differences in live birth and clinical pregnancy rates between the CSD and VD groups were not significant. One possible explanation is that age plays a crucial role in IVF/ICSI-ET, and the powerful influence of age on IVF pregnancy outcomes exceeds that of uterine defects. The success of IVF is highly dependent upon female age. Advanced female age reduces ovarian reserve, decreases oocyte/embryo competence and increases embryonic aneuploidies [26]. Therefore, a uterine defect has only a negligible impact on pregnancy outcomes after IVF compared with advanced age. Our study further assessed the impact of obvious fluid in the niche or fluid in the endometrial cavity on the reproductive outcomes of embryo transfer. The results showed that fluid in the obvious niche or endometrial cavity had no further detrimental effect on implantation compared with nonfluid fluid in the niche cavity. However, the sample size of these subgroups was too small, and larger prospective studies are recommended.

The present study showed that the presence of a caesarean section scar without a defect did not reduce the rates of pregnancy outcomes among IVF cycles. The underlying mechanism that leads to adverse pregnancy outcomes among women with caesarean section after IVF may be attributed to the presence of a scar defect. A more recent review study by Vissers et al. presented a series of hypotheses regarding the effect of a CSD on fertility outcomes [27]. The possible hypotheses relevant to this study are as follows. (1) The first hypothesis that the accumulation of intracavitary fluid or blood related to the defect may impair embryo implantation through embryotoxic factor exposure. Lousse et al. reported that old blood in the uterine cavity may lead to degradation of haemoglobin, resulting in higher iron exposure, which is known to be embryotoxic [28]. (2) The second hypothesis is alterations in immunobiology and/or increased inflammation when a defect is present. A study by Ben-Nagi et al. showed that the major alterations at the scar site were less vascularization and fewer leucocytes than in the endometrium of the unscarred uterus [29]. In addition, Moreno et al. suggested that the accumulation of fluid may promote bacterial growth, reducing the success of IVF [30]. (3) The third hypothesis is uncoordinated or impaired uterine contractions after caesarean section. The site of a previous caesarean scar is discontinuous. The uterine incision is generally in the lower uterine segment and can lead to poor contractility of the uterine muscle around the scar [31]. (4) The fourth hypothesis suggests that ET becomes problematic due to a distorted anatomy resulting from a large defect and a strongly retroflexed uterus. Several studies reported that ET in women with a history of a prior caesarean section took a longer time and was more difficult than ET in women with a prior vaginal delivery [12, 14, 24]. In addition, our study revealed that the endometrial thickness on the trigger day in the CSD group was significantly thinner (9.87 ± 1.74 *vs* 9.72 ± 1.82 *vs* 9.28 ± 1.50, *P* = 0.026). This implied that CSD may also impair implantation by affecting the growth of the endometrium. However, this finding needs further investigation with larger sample size.

Previous studies have provided reliable evidence that women who become pregnant through assisted reproductive technology are more likely to choose caesarean section delivery, which increases the caesarean section rate [32, 33]. In our study, although the previous delivery mode was vaginal in the VD group, the caesarean section rate after IVF/ICSI-ET was 54.1%; however, in the CS and CSD groups, the caesarean section rates were 95.8 and 93.8%, respectively. One possible reason for this finding lies in the increased incidences of obstetrical and perinatal complications that increase the probability of needing a caesarean section [34]. Other possible reasons were that the high risk of IVF pregnancy, the “precious baby” effect and maternal preference in the absence of medical indications play important roles in the decision [35]. In addition, it needs to be pointed out that the high twin birth rate after IVF treatment is also one of the reasons for the increase in the caesarean section rate. Our research also showed that the preterm birth rate in the CSD group was 31.3%, which was higher than the rates in the VD and CS groups (15.8 and 10.9%, respectively). To avoid obstetric complications during the late stage of pregnancy, some patients with a CSD must terminate their pregnancy before a term birth. However, due to the limited sample size, the difference in late-term terminations of pregnancy among the groups was not significant. Due to the development of obstetric-related techniques, none of the women in the three groups had postpartum haemorrhage or uterine rupture.

One strength of our study was that we investigated the role of a niche (caesarean scar defect) in pregnancy outcomes separately, which has not been assessed in previous studies. The reason for the inconsistent conclusion regarding the effect of a CS on pregnancy outcome may be due to the different proportions of women with a CSD in previous studies. However, several limitations should also be noted. First, the overall sample size of the study was still limited, which reduced the statistical power. Post hoc power analysis was carried out based on the number of patients enrolled with live birth rates of 36.4 and 21.6% among the VD and CSD groups, respectively. Let alpha be 0.05; then, the sample of the current study achieves only a power of 72% to detect the difference in live births between these two groups. Second, there were differences in baseline characteristics among the three groups. However, the results remained robust after we adjusted potential confounding factors, including age, infertility cause, infertility category, BMI, endometrial thickness on the trigger day, fertilization methods, number of embryos transferred, number of high-quality embryos transferred and day of transfer in the logistic regression models.Third, we did not collect detailed information regarding the patient’s caesarean section incision types. The relationship between types of previous caesarean sections and the pregnancy outcomes of IVF needs further study.

## Conclusion

This study demonstrated that the existence of a caesarean scar without a niche did not decrease the rates of pregnancy outcomes after IVF/ICSI-ET. However, a uterine niche after caesarean section may have a detrimental effect on subsequent pregnancy. In young women in particular (age ≤ 35 years), the harmful effects of a CSD are more significant. However, in advanced-aged women, age had a more critical effect on pregnancy outcomes. Uterine defects have only a negligible impact on pregnancy outcomes after IVF compared with advanced age. We suspect that the existence of a CSD may be the main reason for the decrease in pregnancies among women with a history of caesarean section. Findings from this study should be of practical clinical importance for counselling women on the potential influence of a previous caesarean section scar with or without a niche on pregnancy outcomes following IVF/ICSI-ET treatment. Clinicians should pay attention to the examination and assessment of caesarean scar defect before IVF. In addition, vigorously promoting vaginal delivery and reducing unindicated caesarean sections at the primary delivery are fundamental measures to reduce the caesarean section rate and its complications. Since the number of patients enrolled in our study was limited, further studies on a larger scale are needed to confirm our conclusions.

## Data Availability

The data used or analyzed during the current study are included within the article. The datasets are not publicly available due to the hospital policy and personal privacy. However, the datasets are available from the corresponding author on reasonable request.

## Abbreviations

IVF/ICSI-ET: In vitro fertilization/intracytoplasmic sperm injection-embryo transfer
VD: Vaginal delivery
CS: Caesarean scar
CSD: Caesarean section defect
COH: Controlled ovarian hyperstimulation
hCG: Human chorionic gonadotropin
TVOR: Transvaginal oocyte retrieval
FSH: Follicle-stimulating hormone
BMI: Body mass index
OR: Odds ratio
